# Functional analysis of human and chimpanzee promoters

**DOI:** 10.1186/gb-2005-6-7-r57

**Published:** 2005-07-01

**Authors:** Florian Heissig, Johannes Krause, Jaroslaw Bryk, Philipp Khaitovich, Wolfgang Enard, Svante Pääbo

**Affiliations:** 1 Max Planck Institute for Evolutionary Anthropology, Deutscher Platz 6, D-04103 Leipzig, Germany

## Abstract

**Background:**

It has long been argued that changes in gene expression may provide an additional and crucial perspective on the evolutionary differences between humans and chimpanzees. To investigate how often expression differences seen in tissues are caused by sequence differences in the proximal promoters, we tested the expression activity in cultured cells of human and chimpanzee promoters from genes that differ in mRNA expression between human and chimpanzee tissues.

**Results:**

Twelve promoters for which the corresponding gene had been shown to be differentially expressed between humans and chimpanzees in liver or brain were tested. Seven showed a significant difference in activity between the human promoter and the orthologous chimpanzee promoter in at least one of the two cell lines used. However, only three of them showed a difference in the same direction as in the tissues.

**Conclusion:**

Differences in proximal promoter activity are likely to be common between humans and chimpanzees, but are not linked in a simple fashion to gene-expression levels in tissues. This suggests that several genetic differences between humans and chimpanzees might be responsible for a single expression difference and thus that relevant expression differences between humans and chimpanzees will be difficult to predict from cell culture experiments or DNA sequences.

## Abstract 2

Twelve promoters of genes differentially expressed between humans and chimpanzees were tested for expression activity in culture cells. Seven promoters showed a significant difference in expression level between the human and chimpanzee promoter, but only three were in the same direction as the tissues, indicating that relevant expression differences between humans and chimpanzees will be difficult to predict from cell culture experiments or DNA sequences

## Background

Thirty years ago, King and Wilson [1] proposed that phenotypic differences between humans and chimpanzees are mainly caused by quantitative changes in gene expression rather than by structural changes in gene products. This idea is promoted also in some reviews [2] and seems to be supported by recent studies [3-8], which show that as many as 10% of all genes expressed in the brain differ in their expression levels between humans and chimpanzees. However, a causative connection between phenotypic differences and gene-expression differences in the two species remains to be established [9]. Similarly, the molecular basis of gene-expression differences between the two species is largely unknown.

The regulation of gene expression is a complex process involving chromatin structure, DNA methylation, transcription initiation, alternative splicing, RNA degradation, translational control, and posttranslational modifications [10,11]. However, initiation of transcription is thought to be a major factor determining the level of gene expression in most systems [12,13]. Studies in maize, yeast, mice, rats and humans indicate that both *cis*- and *trans*-acting factors are involved in transcriptional regulation [14-21]. Although *trans*-acting factors are clearly important, allelic DNA sequence variation in several human promoters has been shown to profoundly influence transcriptional activity [22-26]. Furthermore, the only functional comparison of a human and a chimpanzee promoter published to date shows that three nucleotide differences can lead to large differences in promoter activity [27].

To estimate what fraction of mRNAs differently expressed between human and chimpanzee tissues may be caused by DNA sequence differences in core promoters, we analyzed the activity of human and chimpanzee promoters from 12 genes that differ in their mRNA expression between the two species in brain and liver as measured by microarrays [6]. In each case, 2 kilobases (kb) of the putative human and chimpanzee promoter regions were cloned and tested for their ability to drive the transcription of a reporter gene during transient expression in human cervical carcinoma and neuroblastoma cell lines. The results show that no simple relationship exists between *in vitro *promoter activity and mRNA levels in tissues of the organisms.

## Results

Gene-expression data measured with Affymetrix U95A arrays from livers and the prefrontal cortex of the brains of three humans and three chimpanzees [6] were used to identify genes that differ significantly in expression between the species. To avoid the influence of sequence differences on the hybridization of chimpanzee transcripts to microarray probes designed for human transcripts, we excluded all probes showing inconsistent hybridization patterns in the two species as described elsewhere [8]. The genes were required to be differentially expressed in at least one of the two tissues with a magnitude of at least 1.4-fold (false-discovery rate < 1%). The 71 genes that satisfied these criteria were further selected on the basis of the availability of an annotated transcription start site [28], the availability of human and chimpanzee DNA sequence of high quality, the possibility to place primers for amplification of the promoters as well as the successful amplification and cloning of the promoter fragments (see Materials and methods).

This left us with human and chimpanzee promoters from 12 genes. From each promoter, a fragment covering approximately 1,500 base pairs (bp) upstream and ≤ 500 bp downstream of the transcription start site without including the start codon was cloned in a plasmid in front of a firefly luciferase reporter gene. For each species, three independent clones were isolated and the insert of each clone was sequenced in its entirety. Each plasmid was mixed with a plasmid containing a sea-pansy luciferase reporter gene under the control of a constitutive promoter and transfected into a human neuroblastoma cell line and a human cervical carcinoma cell line, respectively. Experiments were performed in triplicate and the activities of the two luciferases were measured. All constructs showed an activity at least ninefold higher than the promoter-less control vector in at least one of the two cell lines. To control for transfection efficiency, the measurement of the firefly luciferase was normalized to the measurement of the sea-pansy luciferase and the difference in activity between the three human clones and the three chimpanzee clones analyzed.

**Figure 1:**
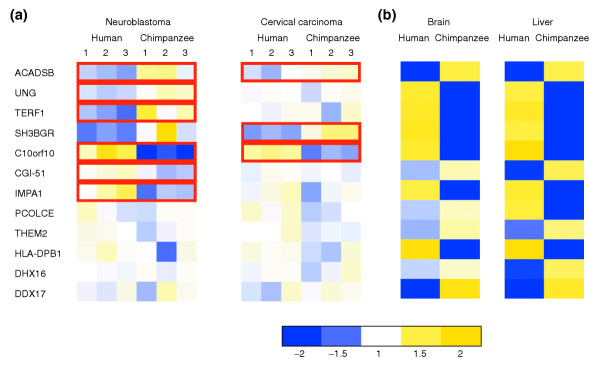
Human and chimpanzee promoter activity and mRNA expression in tissues. **(a) **Promoter activity in cell cultures. Normalized activities of promoters of the indicated genes are compared to the average of the three human and the three chimpanzee clones for each cell line and promoter indicated. Blue indicates lower activity than average, whereas yellow indicates higher activity. The color scale for the fold-change is below. Significant differences in activity between the two species are indicated by red frames. **(b) **Expression of the genes in tissues as assayed by mRNA levels measured by oligonucleotide arrays for brain and liver [6]. The values are averages of the three human and three chimpanzee individuals for which expression levels were determined [6] and are compared to the average level of the corresponding gene.

The results are summarized in Figure 1 and Table 1. Out of the 12 promoters tested, two (ACADSB, C10orf10) show a significant difference (ANOVA *p*-value < 0.05) in both cell lines whereas five (IMPA1, CGI-51, SH3BGR, UNG, TERF) show a significant difference in only one of the two cell lines. Five promoters show no significant difference in either cell line. The average sequence divergence (Table 1) for these five promoters (1.2%) and the remaining seven (1.3%) is not significantly different from each other, neither for the complete promoter fragment (two-tailed *t*-test, p = 0.65) nor for 220 bp around the transcription start site (*p *= 0.43) in which most of the conserved regulatory motifs are found [29]. One promoter (THEM2) contains a chimpanzee-specific *Alu *insertion, but does not show a significant difference in its activity in either of the two cell lines.

Three promoters (ACADSB, C10orf10, IMPA1) show activity differences in the promoter assays that go in the same direction as the expression differences of the corresponding genes in the tissues. Interestingly, the two promoters (ACADSB, C10orf10) that show qualitatively similar differences in the two cell lines are both in concordance with the tissue expression differences. For four promoters (CGI-51, SH3BGR, UNG, TERF) that show differences in only one of the cell lines, the difference goes in the opposite direction to the expression differences in the tissues.

## Discussion

We have compared the transcriptional activity of human and chimpanzee promoters from 12 genes that differ in their expression levels between humans and chimpanzees in tissues. We find that seven of the 12 promoter pairs differ significantly in their transcriptional activity in at least one of the two cell lines used (Figure 1, Table 1). This is in agreement with the finding that many proximal promoters that show sequence differences among humans differ in their activity in promoter assays [22,25,26,30]. Furthermore, we find that in five cases promoter activity differences are restricted to one of the two cell lines, showing that interspecies differences in promoter activity are often specific to cell line or tissue, an observation that is compatible with previous work on allelic promoter differences among different human cell lines [22,26] and tissue-specific *cis-*acting variation in mice [31] and humans [32]. Clearly, in order to predict such differences in promoter activity from their DNA sequences, much more knowledge on the occurrence of transcription factors in cells and their binding sites *in vivo *is needed.

What might seem more unexpected is that the differences in promoter activity observed in the cell lines seem to be independent of the differences in expression seen in the tissues. The transcript levels of only three genes (ACADSB, C10orf10, IMPA1) go in the same direction in at least one of the cell lines as they do in the tissues, whereas they go in the opposite direction for four genes (CGI-51, SH3BGR, UNG, TERF) in one of the two cell lines. One possible explanation is that the expression differences observed in tissues are due to differences in environmental factors between the species. This, however, seems unlikely, given the high reproducibility of expression differences between studies [5,33] that use different individuals. Assuming that the observed expression differences in tissues are indeed genetic in nature, one possibility to account for the discrepancy with the promoter activities observed *in vitro *is that the same sequence differences in the proximal promoters have opposite effects in different tissues or cell lines. However, none of the 12 promoters tested showed a significant opposite effect in the two cell lines (Figure 1), nor did any of 43 allelic variants of human promoters tested in a previous study show an opposite effect in different cell lines [22,26]. Furthermore, over 99% of the genes that show a significant expression difference between humans and chimpanzees in two tissues show the same direction of change in the two tissues [6,34]. Thus, it seems unlikely that a tissue- or cell-type effect is responsible for the opposite expression patterns seen here between the cell lines and the tissues.

A remaining possibility is that additional genetic differences between humans and chimpanzees outside the proximal promoters are numerous and/or strong enough to lead to gene-expression levels in tissues that are often qualitatively opposite to what would be inferred from the activity of the proximal promoter *in vitro*. This agrees with the observation that many gene-expression differences are inherited as quantitative traits, that is, several genetic loci are responsible for allelic gene expression differences observed among humans and among mice [14,21,35]. In fact, when gene-expression differences between humans and chimpanzees are compared, the number of loci affecting the expression of single genes is likely to be even higher than for allelic differences, as these two species are more diverged than individual mice or humans. Of possible relevance in this respect is the recent finding that promoters are much less conserved, relative to intronic regions, between human and chimpanzee than between mouse and rat [36]. A probable cause of this is the smaller effective population size of primates than of rodents, which would have allowed slightly deleterious regulatory variants to become fixed in periods when the population size was small [36]. When the population size, and hence the effectiveness of selection, subsequently increased, compensatory mutations outside the proximal promoters might have become fixed. An example of such compensatory mutations has been described for the *even-skipped *stripe 2 enhancer in two *Drosophila *species [37].

It is worth noting that the genes studied here are all differentially expressed in human and chimpanzee tissues. It is still unclear to what extent the discordance between behavior of promoters *in vitro *and the corresponding tissue mRNA levels also holds true for genes that do not show an expression difference between human and chimpanzee tissues. If many genetic differences do indeed influence the expression of a single gene, the proximal promoters of these non-differentially expressed genes would be expected to differ in their activity almost as frequently as the promoters of differentially expressed genes.

Our results imply that although many promoters may differ in activity between humans and chimpanzees, it will be difficult to predict physiologically relevant gene-expression differences from promoter activities observed in cell lines, even between two closely related species such as humans and chimpanzees. Further work is necessary to elucidate to what extent this applies also to allelic DNA sequence differences in promoters observed within a species. Further work is also needed to elucidate whether a general paradigm for how genome structure translates to gene expression activity can be derived.

## Materials and methods

### Selection of promoters for study

Genes for promoter analysis were selected on the basis of a large-scale transcriptome comparison between three humans and three chimpanzees in brain and liver using Affymetrix HG U95A and HG U95Av2 arrays [6]. All microarray analysis was performed using the MAS 5.0 software package from Affymetrix. Selected genes were required to be differentially expressed in at least one of the two tissues with average change *p*-value < 0.05 or > 0.95 (two-sided test) and with a fold-change magnitude of at least 1.4-fold. The false-discovery rate was determined by applying these selection criteria to 10,000 permutations of the original dataset with randomly assigned sample labels. Expression differences were confirmed by masking all probes showing inconsistent hybridization patterns in the two species using a custom mask file as described elsewhere [8].

We used the DBTSS databases of transcriptional start sites [38] (August 2003) to identify the transcription start sites and to collect promoter sequences 2,000 bp upstream and 1,000 bp downstream of the start sites. Out of a total of 71 genes satisfying the gene-expression-based criteria (see Additional data file 1), 35 had annotated transcription start sites and chimpanzee sequence was available (July 2003). For 24 of them, primers were designed using Primer 3 software [39] to amplify approximately 1,500 bp upstream and 500 bp downstream of the transcription start site, if possible without including any coding sequence or the start codon. Restriction sites for *Bgl*II or *Xho*I (depending on the presence of restriction sites in the promoter sequence) were added to the 5'-end of the primer for cloning. Using these primers (see Additional data file 2), we were able to amplify and isolate three independent clones from both species for 12 genes.

### Cloning and reporter gene assay

DNA from one human and one chimpanzee was amplified using Expand 20 kb Plus PCR system (Roche) according to the manufacturer's protocols with an extension time of 3 min, or *Pfu *DNA polymerase (Stratagene), using the following conditions: 1 min at 96°C, 45 sec at 96°C, 45 sec at 61°C, 5 min at 72°C for 38 cycles, followed by a final extension at 72°C for 10 min, on Tetracyclers (MJ Research).

PCR products were purified up by QIAquick eight-well cleanup kit (Qiagen), digested with either *Bgl*II (NEB) or *Xho*I (NEB), purified on 1% low-melting agarose gels (Promega) and isolated using QIAquick gel purification kit (Qiagen). These fragments were cloned upstream of the firefly luciferase gene into the *Bgl*II or *Xho*I site of the pGl3 vector (Promega), using T4 Quick Ligase (NEB) and One Shot Top 10 F cells (Invitrogen). Colonies were picked and heated in 10 μl water for 5 min at 96°C, and 2 μl was used as template in a 25 μl PCR reaction, using one primer in the vector (GL2) and one primer in the promoter.

Positive clones were grown in 7 ml LB medium (Invitrogen) containing 100 μg/ml ampicillin (Sigma) at 37°C overnight. Vector DNA was isolated using a Miniprep kit (Qiagen), and DNA concentration was measured on a Nanodrop UV spectrophotometer (NanoDrop Technologies). All inserts were sequenced (Additional data file 4) using Big Dye Terminator chemistry (Applied Biosystems).

The human neuroblastoma cell line (SHEP [27]) was obtained from Martin Reick, University of Texas Southwestern Medical Center, and the human cervical carcinoma cell line (c33a) (ATCC Number HTB-31) was obtained from Kurt Engeland, University of Leipzig. SHEP cells were grown in DMEM (Gibco) medium supplemented with 15% fetal bovine serum (Sigma) and c33a cells in DMEM/MIX F12 (Gibco) supplemented with 10% fetal bovine serum (Sigma), and plated at ~85% confluence a day before transfection. One microgram of the promoter constructs was mixed with 67.4 ng of the pRL-SV40 vector (Promega) containing the sea-pansy luciferase gene in 96.8 μl serum-free medium (Optimem1, Gibco) and 2.5 μl Lipofectamine 2000 (Gibco). Cells were transfected in triplicate for 4 h at 37°C, 5% CO_2 _and 100% humidity, grown for 20 h and then lysed in 100 μl lysis buffer (Promega).

A 5 μl sample of lysate was used in a Dual-Luciferaser Reporter Assay System (Promega) in a Wallac Victor 2 Luminometer (PerkinElmer). Promoter activity was measured by normalizing the luciferase activity of the promoter constructs to the sea-pansy luciferase activity of the control plasmid from the same well (see Additional data file 3). We assessed the significance (p < 0.05) of different activity in human and chimpanzee promoters using a multi-way ANOVA including species, clones, and replicates.

## Additional data files

Additional data is available with the online version of this paper. Additional data file 1 is a table listing the 71 genes differentially expressed between humans and chimpanzees in liver and/or brain. For the probe sets the change *p*-values (MAS 5.0) averaged over the 36 pairwise comparisons between human and chimpanzee samples [6] and the signal log_2 _ratio (slr; MAS 5.0) for the probe sets are given. *p*-values close to 1 suggest a higher expression in chimpanzees, as does a negative slr. Additional data file 2 is a table listing the primers used for promoter amplification. Primer sequences are written 5' to 3' and lower-case letters indicate added restriction sites. Additional data file 3 is a table listing measured promoter activities (the measured luciferase activity of the promoter constructs divided by the sea-pansy luciferase activity, for each of the three replicates that were done for each of the three clones for humans and chimpanzees in the neuroblastoma cell line and the cervix carcinoma cell line, respectively, for the 12 genes analyzed). Additional data file 4 contains the nucleotide sequences of the insert of the used vectors.

## Supplementary Material

Additional File 1 Supplementary Table 1: Listed are all 71 genes that were identified as being at least 1.4-fold differently expressed in liver and/or brain. For the probe sets the change *p*-values (MAS 5.0) averaged over the 36 pairwise comparisons between human and chimpanzee samples [6] and the signal log_2 _ratio (slr; MAS 5.0) for the probe sets are given. *p*-values close to 1 suggest a higher expression in chimpanzees, as does a negative slr. Click here for file

Additional File 2 Supplementary Table 2: The primer sequences used to amplify the promoter fragments are given here. Primer sequences are written 5' to 3' and lower-case letters indicate added restriction sites. Click here for file

Additional File 3 Supplementary Table 3: The normalized promoter activities are listed; that is, the measured luciferase activity of the promoter constructs divided by the sea-pansy luciferase activity, for each of the three replicates that were done for each of the three clones for humans and chimpanzees in the neuroblastoma cell line and the cervix carcinoma cell line, respectively, for the 12 genes analyzed. Click here for file

Additional File 4 The nucleotide sequences of the cloned promoters. Click here for file

## Figures and Tables

**Table 1:** DNA sequence and expression divergence of human and chimpanzee promoters

Promoter	Divergence^†^	Divergence TSS^‡^	Brain^§^	Liver^§^	Neuroblastoma^¶^	Cervical carcinoma^¶^
ACADSB	1.4	3.3	-1.6	-1.3	-0.5*	-0.3*
C10orf10	1.3	1.8	ND	4.0	1.4**	0.7***
CGI-51	1.6	1.9	-0.4	-1.3	0.3*	0.1
DDX17	1.8	2.0	-4.2	-4.4	0	-0.1
DHX16	1.5	0.9	-0.8	-1.7	0	0.1
HLA-DPB1	0.8	0.9	2.5	4.1	0.2	0.2
IMPA1	1.5	2.6	2.3	2.6	0.6*	0.2
PCOLCE	0.9	0.6	ND	2.1	0	0.2
SH3BGR	1.6	1.3	1.9	2.4	-0.8	-0.7***
TERF1	1.5	1.4	2.3	1.6	-0.7*	0
THEM2	1.2	2.6	-0.5	-0.8	0.1	0.1
UNG	0.6	0.5	2.5	1.8	-0.3*	0

^†^Point substitutions per compared site in the whole promoter (~ 2 kbp); ^‡^point substitutions per compared site around the transcription start site (-200 to +20); ^§^signal log_2 _ratio as determined from arrays, where positive values indicate higher expression in humans (that is, a value of 1 means twofold higher expression in humans); ^¶^average signal log_2 _ratio for the promoter activity in cell lines; *, ** and *** indicate *p*-values (ANOVA) < 0.05, 0.01, and 0.001, respectively.
